# Association Between Ambient Heat and Risk of Emergency Department Visits for Mental Health Among US Adults, 2010 to 2019

**DOI:** 10.1001/jamapsychiatry.2021.4369

**Published:** 2022-02-23

**Authors:** Amruta Nori-Sarma, Shengzhi Sun, Yuantong Sun, Keith R. Spangler, Rachel Oblath, Sandro Galea, Jaimie L. Gradus, Gregory A. Wellenius

**Affiliations:** 1 Department of Environmental Health, Boston University School of Public Health, Boston, Massachusetts; 2 OptumLabs Visiting Scholar, Eden Prairie, Minnesota; 3 Department of Psychiatry, Boston Medical Center, Boston, Massachusetts; 4 Boston University School of Public Health, Boston, Massachusetts; 5 Department of Epidemiology, Boston University School of Public Health, Boston, Massachusetts

## Abstract

**Importance:**

The implications of extreme heat for physical health outcomes have been well documented. However, the association between elevated ambient temperature and specific mental health conditions remains poorly understood.

**Objective:**

To investigate the association between ambient heat and mental health–related emergency department (ED) visits in the contiguous US among adults overall and among potentially sensitive subgroups.

**Design, Setting, and Participants:**

This case-crossover study used medical claims data obtained from OptumLabs Data Warehouse (OLDW) to identify claims for ED visits with a primary or secondary discharge psychiatric diagnosis during warm-season months (May to September) from 2010 through 2019. Claims for adults aged 18 years or older with commercial or Medicare Advantage health insurance who were living in 2775 US counties were included in the analysis. Emergency department visits were excluded if the Clinical Classifications Software code indicated that the visits were for screening for mental health outcomes and impulse control disorders.

**Exposures:**

County-specific daily maximum ambient temperature on a continuous scale was estimated using the Parameter-Elevation Relationships on Independent Slopes model. Extreme heat was defined as the 95th percentile of the county-specific warm-season temperature distribution.

**Main Outcomes and Measures:**

The daily incidence rate of cause-specific mental health diagnoses and a composite end point of any mental health diagnosis were assessed by identifying ED visit claims using primary and secondary discharge diagnosis *International Classification of Diseases, Ninth Revision* and *International Statistical Classification of Diseases and Related Health Problems, Tenth Revision* codes. Conditional logistic regression models were used to estimate the incidence rate ratio (IRR) and 95% CIs for the association between daily temperature and incidence rates of ED visits.

**Results:**

Data from 3 496 762 ED visits among 2 243 395 unique individuals were identified (56.8% [1 274 456] women; mean [SD] age, 51.0 [18.8] years); of these individuals, 14.3% were aged 18 to 26 years, 25.6% were aged 27 to 44 years, 33.3% were aged 45 to 64 years, and 26.8% were aged 65 years or older. Days of extreme heat were associated with an IRR of 1.08 (95% CI, 1.07-1.09) for ED visits for any mental health condition. Associations between extreme heat and ED visits were found for specific mental health conditions, including substance use disorders (IRR, 1.08; 95% CI, 1.07-1.10); anxiety, stress-related, and somatoform disorders (IRR, 1.07; 95% CI, 1.05-1.09); mood disorders (IRR, 1.07; 95% CI, 1.05-1.09); schizophrenia, schizotypal, and delusional disorders (IRR, 1.05; 95% CI, 1.03-1.07); self-harm (IRR, 1.06; 95% CI, 1.01-1.12); and childhood-onset behavioral disorders (IRR, 1.11; 95% CI, 1.05-1.18). In addition, associations were higher among men (IRR, 1.10; 95% CI, 1.08-1.12) and in the US Northeast (IRR, 1.10; 95% CI, 1.07-1.13), Midwest (IRR, 1.11; 95% CI, 1.09-1.13), and Northwest (IRR, 1.12; 95% CI, 1.03-1.21) regions.

**Conclusions and Relevance:**

In this case-crossover study of a large population of US adults with health insurance, days of extreme heat were associated with higher rates of mental health–related ED visits. This finding may be informative for clinicians providing mental health services during periods of extreme heat to prepare for increases in health service needs when times of extreme heat are anticipated.

## Key Points

**Question:**

Are periods of higher ambient temperature associated with an increase in emergency department (ED) visits for mental health conditions among US adults with health insurance?

**Findings:**

In this case-crossover study of 3 496 762 ED visits among 2 243 395 unique individuals, higher warm-season temperatures were associated with an increased risk of ED visits for any mental health condition and for specific mental health conditions.

**Meaning:**

This information could aid clinicians providing services for mental health in preparing for increased stress on individuals and the health care system during times when extreme heat is anticipated.

## Teaser

This case-crossover study examines the association between higher warm-season temperatures and the number of mental health–related emergency department visits by US adults with health insurance.

## Introduction

Exposure to high ambient temperatures (ie, heat) is a recognized threat to public health and has been documented to be associated with excess morbidity^1^ and mortality.^2,3,4^ Seven of the warmest years on record for the contiguous US have occurred since 2014, with 2016 reaching the greatest temperatures and 2020 now ranked as the second warmest year in the available 141-year record.^5^ As climate change leads to more days with extreme temperatures, and particularly, higher summertime temperatures, the burden of disease associated with ambient heat is expected to increase. Heat stress is known to trigger adverse physiological responses in the human body, ranging from heat rash and muscle cramps or fatigue to broad consequences for a range of human organ systems and heat stroke, which can be fatal.^6^

In addition to the association between extreme heat and physical health, a growing number of studies have reported on the potential adverse effects of heat on mental health. Ambient temperature has been previously associated with exacerbation of symptoms for many mental and behavioral disorders, including self-reported adverse mental health outcomes,^7,8,9^ and elevated risk of emergency department (ED) visits for any mental health cause,^9^ mood-anxiety disorders, substance use, and schizophrenia^10,11^ as well as higher suicide risk.^9,12,13^ However, existing studies have been limited by small sample sizes, specific populations or geographic areas, or reliance on self-reported mental health symptoms. Thus, the association between heat and mental health remains incompletely quantified, and little is known about whether certain population subgroups have increased risk factors for visiting the ED for mental health diagnoses because of exposure to higher ambient temperature.

Mental health consequences of elevated ambient temperature can arise during both warm- and cool-temperature seasons. However, the underlying processes that lead to elevated adverse mental health outcomes may be different by season. For example, cold temperatures may affect health on a different time scale, with substantially longer lag effects during cold periods compared with hot periods.^14,15,16^ In addition, virtually all extreme heat events in the US occur during the warm season. Therefore, although it is important to assess the association between temperature and mental health across the entire year, the proposed statistical method in the current analysis is better suited to a warm-season-only model. The aim of this study was to investigate the association between warm-season (May through September) temperatures between 2010 and 2019 and rates of ED visits for a broad range of mental health outcomes among adults with commercial and Medicare Advantage health insurance living in the contiguous US. We focus on ED visits, which represent the most severe presentations of mental health exacerbations both from a clinical perspective and in terms of stress on health systems to provide care. We further investigated whether observed associations differed across strata defined by age, sex, and geographic region and explored the time course of the observed association.

## Methods

**Figure 1.**
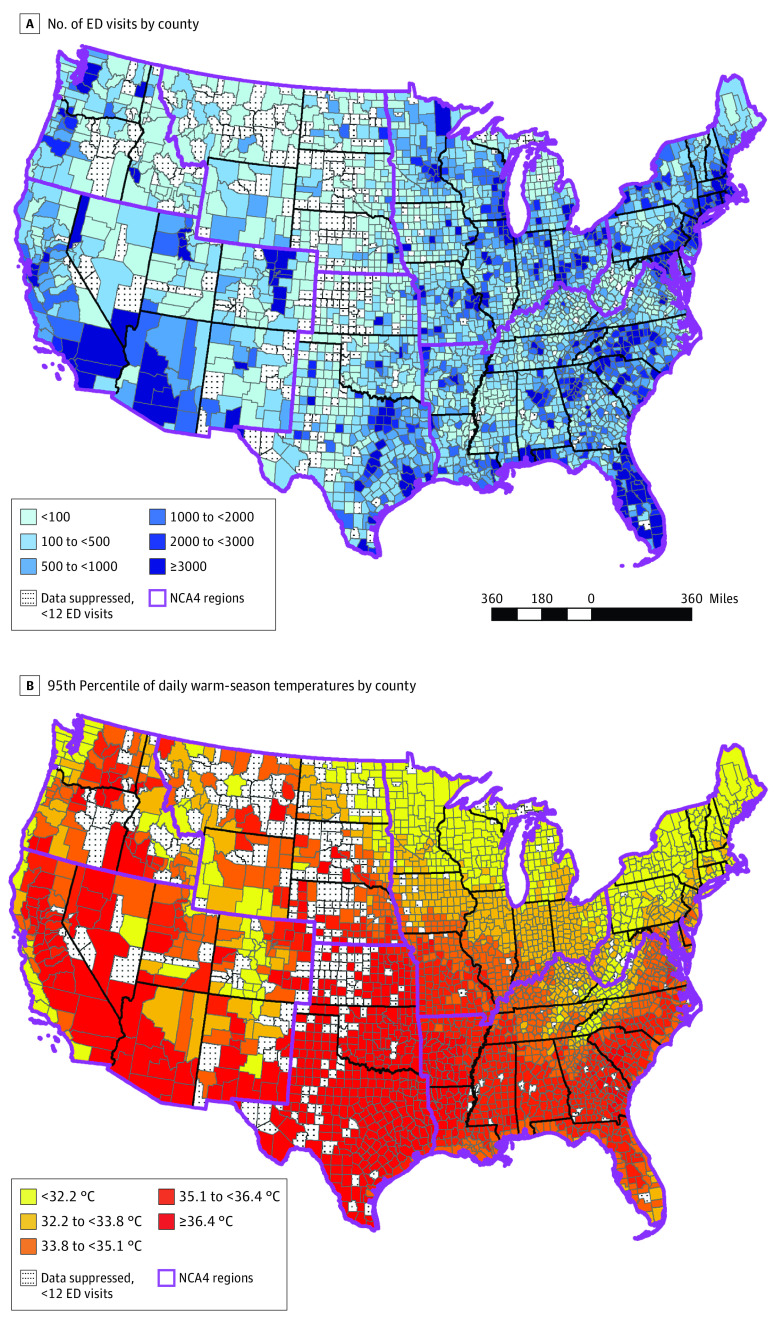
Maps of Contiguous US Showing County-Level Data on Number of Emergency Department (ED) Visits and Warm-Season Temperatures NCA4 indicates Fourth National Climate Assessment.

In this case-crossover study, we obtained medical claims between January 1, 2010, and December 31, 2019, from the OptumLabs Data Warehouse (OLDW), which contains deidentified, longitudinal health information on enrollees and patients, representing a diverse mixture of ages, ethnicities, and geographies throughout the contiguous US.^17^ We identified claims for ED visits related to mental health (Figure 1A) based on the *International Classification of Diseases, Ninth Revision (ICD-9)* or *International Statistical Classification of Diseases and Related Health Problems, Tenth Revision (ICD-10)* code, revenue code, *Current Procedural Terminology* code, and place of service code. For each claim, we then extracted information on the age, sex, and county of residence of the individual as well as the admission date and principal diagnosis code (based on *ICD-9* until 2015 or *ICD-10* after 2015) for each ED visit. Information on race and ethnicity was unavailable in these data sets. We limited our analysis to ED visits occurring among individuals aged 18 years or older. The institutional review board of Boston University deemed the study exempt from review and waived the requirement for informed consent because the study involved analysis of deidentified data. This study followed the Strengthening the Reporting of Observational Studies in Epidemiology (STROBE) reporting guideline.

We applied the Agency for Healthcare Research and Quality’s Clinical Classifications Software scheme^18^ to *ICD-9* and *ICD-10* principal diagnosis codes at discharge, including primary discharge diagnosis and secondary diagnoses, to classify ED visits into clinically meaningful and mutually exclusive disease groups. The Clinical Classifications Software scheme is a comprehensive classification tool for clustering diagnoses into a manageable number of categories based on disease characteristics and treatment protocol and is widely used to analyze disease-specific conditions. We identified the disease groups for relevant mental health outcomes^19^ as specified in Table 1. We excluded the Clinical Classifications Software codes for screening for mental health outcomes because the data-generation process is different than for a diagnosis and may lead to inaccuracies in the data. We further excluded ED visits for impulse control disorders, which are uncommon in this data set.

**Table 1.** Mean Daily Emergency Department (ED) Visits for Mental Health and Specific Causes Across 2775 US Counties, May to September, 2010 to 2019^a^

Reason for ED visit (n = 3 496 762)^b^	CCS code	Mean (SD) ED visits			25th Percentile^c^	50th Percentile	75th Percentile
		Overall	Men	Women			
Mental health	650-652, 654, 655, 657-662, 670	2286 (966)	968 (452)	1318 (516)	1511	1702	3363
Substance use disorders	660, 661	1063 (827)	576 (418)	487 (410)	382	456	2026
Anxiety, stress-related, and somatoform disorders	650, 651	797 (240)	256 (73)	541 (169)	623	767	1001
Mood disorders	657	764 (155)	246 (51)	518 (106)	651	733	877
Schizophrenia, schizotypal, and delusional disorders	659	121 (22)	58 (12)	63 (13)	105	121	136
Self-harm	662	120 (44)	55 (21)	65 (24)	84	107	159
Childhood-onset behavioral disorders	652, 654, 655	88 (19)	45 (11)	43 (10)	77	89	101
Miscellaneous	670	43 (9)	13 (5)	30 (7)	37	43	49
Adult personality and behavior disorders	658	16 (5)	5 (3)	11 (4)	12	15	19

Abbreviation: CCS, Clinical Classifications Software.

^a^ Data source: OptumLabs Data Warehouse (OLDW).

^b^ The eTable in the Supplement includes the corresponding *International Classification of Diseases, Ninth Revision* and *International Statistical Classification of Diseases and Related Health Problems, Tenth Revision* codes.

^c^ The 3 percentiles represent ED visits at the county level, showing the distribution around the mean.

### Assessment of Ambient Temperature

We obtained daily maximum ambient temperature data from the Parameter-Elevation Regressions on Independent Slopes (PRISM) model from the PRISM Climate Group,^20^ which is a validated spatiotemporal model with approximately 4-km horizontal grid spacing.^21^ To represent population exposure to temperature, we calculated a population-weighted mean daily maximum temperature provided by the PRISM model for each day in each county, as described previously in the literature.^22^ We limited the study period to the warm-season months (May through September; henceforth referred to as the warm season for simplicity) to represent heat exposure. We estimated extreme temperature as days with a daily maximum ambient temperature greater than or equal to the 95th percentile of county-specific temperature (Figure 1B). For sensitivity analyses, we also estimated a population-weighted mean daily ambient temperature based on PRISM data.

### Statistical Analysis

We used a case-crossover study^23,24^ to estimate the association between daily maximum temperature and the incidence rate per county-day of ED visits with a diagnosis for a composite end point of any mental health condition and ED visits for specific mental health conditions. In this study design, participants serve as their own control, and the inference is based on the comparison of exposures over time within the same individual. This design has the advantage of controlling for all known and unknown potential confounders that are time invariant or vary relatively slowly over long periods of time (eg, socioeconomic status, age, and sex). We used a time-stratified approach to select control periods such that ambient temperature during the case period was compared with ambient temperature on other days of the same year, month, and day of the week as the case day.^25,26^ This approach to selecting control periods serves to minimize confounding by seasonal and long-term time patterns as well as day of the week.^25^ In addition, we adjusted for relative humidity (natural spline with 3 *df*) and federal holidays.

In the primary analysis, we applied a well-established distributed lag nonlinear modeling framework to allow for both nonlinear exposure-response functions and nonlinear lag-response functions.^27,28^ We modeled exposure-response functions using a quadratic B-spline, with 1 internal knot placed at the 50th percentile of county-specific warm-season months’ temperature distribution. For the lag-response function, we used a natural cubic B-spline with 2 knots placed at equal intervals on the log scale of lags up to 5 days. We used conditional logistic regression models to estimate the incidence rate ratio (IRR) and 95% CIs for the association between daily temperature and incidence rates of ED visits, comparing ED visits associated with ambient temperature with ED visits associated with the optimal temperature. The optimal temperature was estimated as the temperature percentile with minimum ED visits across the county-specific temperature distribution. Extreme heat was defined as ambient temperature at the 95th percentile of the county-specific temperature distribution. We first considered the association between temperature and the IRR of ED visits associated with a composite end point of any mental health condition. We subsequently considered the association between temperature and the IRR of ED visits for specific mental health conditions.

We performed a series of sensitivity analyses using the composite mental health end point to assess the robustness of our findings. First, we varied the key modeling parameters to estimate the association between ambient heat and ED visits for the composite mental health end point. This sensitivity analysis included exposure-response functions using a quadratic B-spline with 2 and 3 internal knots. We modeled the lag-response function using a natural cubic B-spline with 3 knots placed at equal intervals on the log scale of lags up to 5 days. Second, because there is no consensus on which exposure metrics should be used to examine the impact of heat, we used daily mean temperature in the sensitivity analysis.

To examine differences in the rate of ED visits for population subgroups, we evaluated whether the association between warm-season heat and incidence of ED visits varied across strata defined by age, sex, and region in the US (defined using the Fourth National Climate Assessment^29^ regions). We used the Wald test to assess whether the associations were homogeneous across strata.^30^

We conducted all analyses in R software, version 3.6.3 (R Foundation for Statistical Computing), with the survival package, version 3.2-7, for the conditional logistic regression and the dlnm package, version 2.4.2, for the distributed lag nonlinear model.

## Results

Between 2010 and 2019, we identified 3 496 762 claims for ED visits occurring among 2 243 395 unique individuals (56.8% [1 274 456] women and 43.2% [968 939] men; mean [SD] age, 51.0 [18.8] years); of these individuals, 14.3% were aged 18 to 26 years, 25.6% were aged 27 to 44 years, 33.3% were aged 45 to 64 years, and 26.8% were aged 65 years or older. This sample represented claims for mental health conditions among 21 048 502 individuals (approximately 6.8% of the 2015 US population) enrolled in commercial or Medicare Advantage health insurance plans. Emergency department visits for substance use disorders were most common, followed by ED visits for anxiety, stress-related, and somatoform disorders and for mood disorders (Table 1). The individuals included in this analysis resided in 1 of 2775 US counties; these counties are the most populated areas within the contiguous US, accounting for locations where approximately 97.6% of the 2020 US population (331 449 281 people) resided.

**Figure 2.**
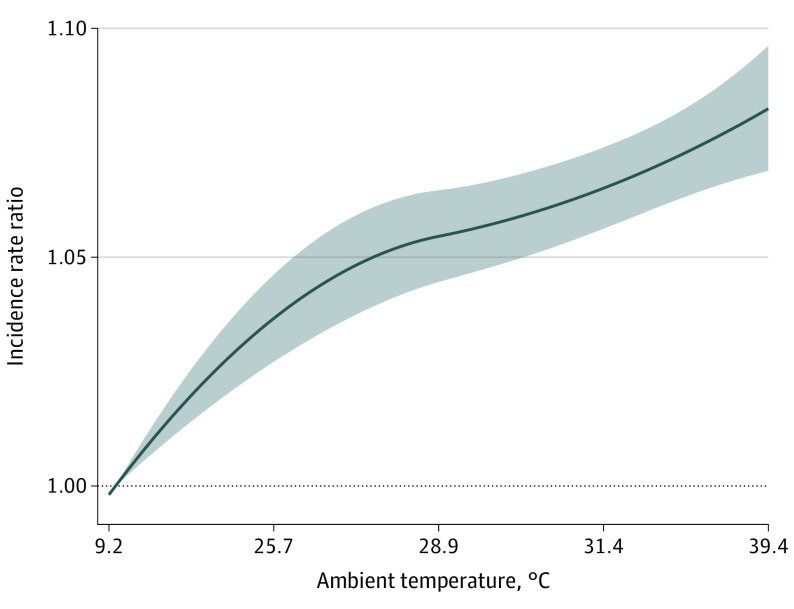
Cumulative Exposure-Response Curve of the Association Between Warm-Season Temperatures and Emergency Department Visits for Any Mental Health Condition Incidence rate ratio of emergency department visits with increasing temperature compared with optimal temperature. Main model adjusted for relative humidity and day of the week. Shading represents the 95% CI. The optimal temperature is the first percentile of the county-specific temperature distribution, at which minimum morbidity occurs. The additional temperatures shown on the x-axis represent the 25th, 50th, 75th, and 100th percentiles of the county-specific temperature distribution, converted to the equivalent actual temperature across all counties in the study area.

**Figure 3.**
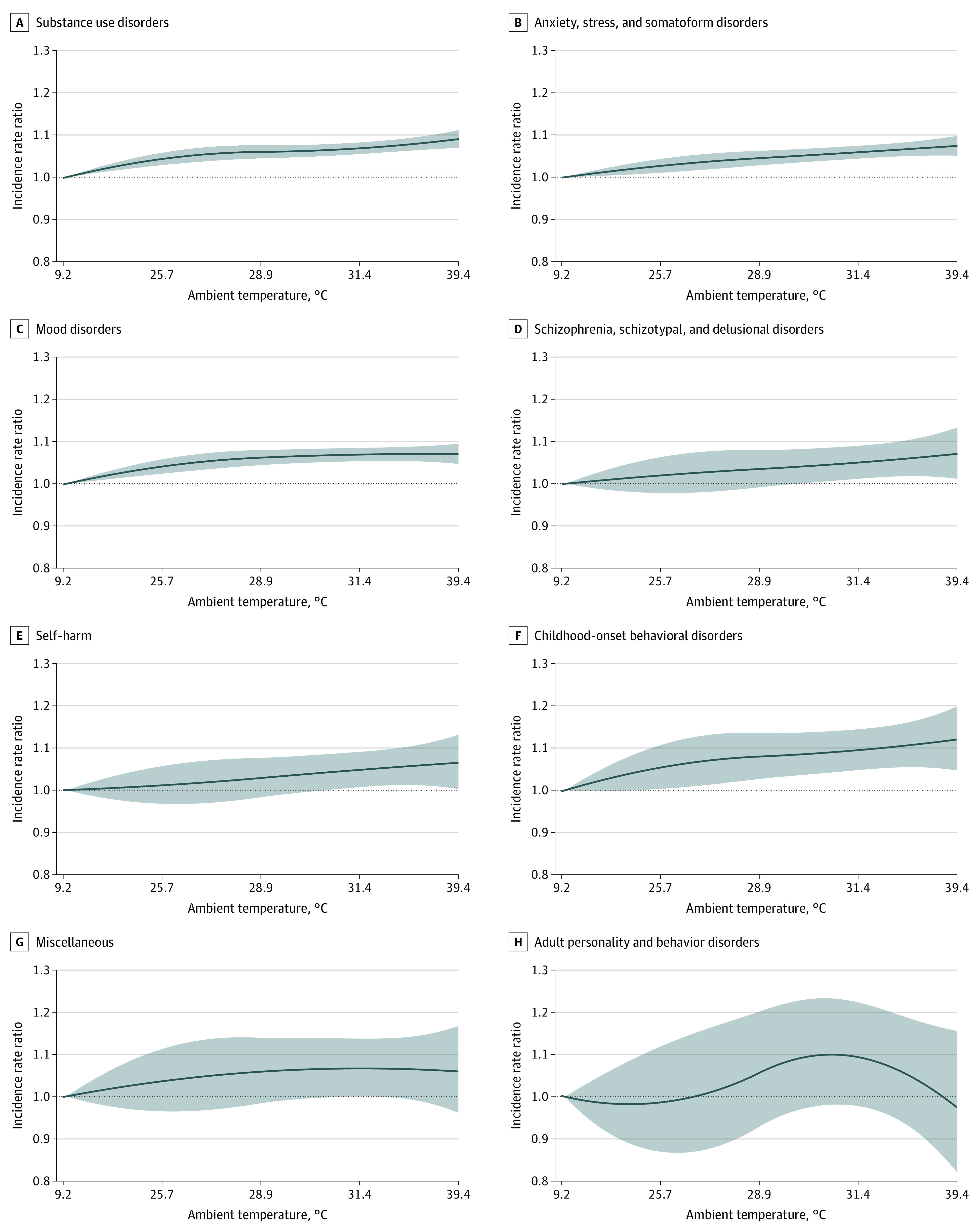
Cumulative Exposure-Response Curve of the Association Between Warm-Season Temperatures and Emergency Department Visits for Specific Mental Health Conditions Incidence rate ratio of emergency department visits with increasing temperature compared with optimal temperature. Main model adjusted for relative humidity and day of the week. Shading indicates the 95% CI. The optimal temperature is the first percentile of the county-specific temperature distribution, at which minimum morbidity occurs. The additional temperatures shown on the x-axis represent the 25th, 50th, 75th, and 100th percentiles of the county-specific temperature distribution, converted to the equivalent actual temperature across all counties in the study area.

Overall, higher warm-season temperatures were associated with monotonically higher rates of ED visits for any mental health condition (Figure 2). Specifically, days of extreme heat had an IRR of 1.08 (95% CI, 1.07-1.09) for ED visits for any mental health condition compared with days of optimal temperature. The increase in IRR was highest on the same day (lag 0), with some evidence of continued higher IRR 2 to 4 days later (eFigure 1 and eAppendix in the Supplement). This result was robust to sensitivity analysis incorporating various modeling parameters (eFigure 2 in the Supplement). Days of extreme heat were also associated with higher rates of ED visits for specific mental health conditions, including substance use disorders (IRR, 1.08; 95% CI, 1.07-1.10); anxiety, stress-related, and somatoform disorders (IRR, 1.07; 95% CI, 1.05-1.09); mood disorders (IRR, 1.07; 95% CI, 1.05-1.09); schizophrenia, schizotypal, and delusional disorders (IRR, 1.05; 95% CI, 1.03-1.07); self-harm (IRR, 1.06; 95% CI, 1.01-1.12); and childhood-onset behavioral disorders (IRR, 1.11; 95% CI, 1.05-1.18) (Table 2). The association between higher temperatures and mental health was less evident for other specific mental health conditions, including adult personality and behavior disorders and other miscellaneous disorders that are not otherwise classified (Figure 3). There was no evidence of lag effects of temperature for specific causes (eFigure 3 in the Supplement). We evaluated how the observed associations between higher temperature and ED visits for any mental health condition varied by age, sex, and geographic region within the US (eFigure 4 in the Supplement). We found no evidence of heterogeneity across age groups but found elevated rates of ED visits for mental health among men (IRR, 1.10; 95% CI, 1.08-1.12) compared with women (IRR, 1.06; 95% CI, 1.05-1.08). We also found that IRRs were higher in the Northeast (IRR, 1.10; 95% CI, 1.07-1.13), Midwest (IRR, 1.11; 95% CI, 1.09-1.13), and Northwest (IRR, 1.12; 95% CI, 1.03-1.21) US.

**Table 2.** Comparison of Overall and Cause-Specific Emergency Department Visits During Periods of High Temperature vs Optimal Temperature

Reason for ED visit	Incidence rate ratio (95% CI)	
	95th Percentile of local warm-season maximum daily temperature	80th Percentile of local warm-season maximum daily temperature
Overall	1.08 (1.07-1.09)	1.07 (1.06-1.08)
Substance use disorders	1.08 (1.07-1.10)	1.07 (1.06-1.08)
Anxiety, stress-related, and somatoform disorders	1.07 (1.05-1.09)	1.06 (1.05-1.08)
Mood disorders	1.07 (1.05-1.09)	1.07 (1.05-1.09)
Schizophrenia, schizotypal, and delusional disorders	1.05 (1.03-1.07)	1.05 (1.02-1.09)
Self-harm	1.06 (1.01-1.12)	1.05 (1.01-1.09)
Childhood-onset behavioral disorders	1.11 (1.05-1.18)	1.10 (1.05-1.15)
Miscellaneous	1.06 (0.98-1.15)	1.07 (1.00-1.14)
Adult personality and behavior disorders	1.01 (0.87-1.17)	1.08 (0.97-1.21)

## Discussion

In this nationwide study of ED visits among adults with commercial and Medicare Advantage health insurance in the contiguous US, we found that days of extreme heat were associated with higher rates of ED visits for a composite measure of mental health diagnoses and ED visits associated with specific mental health conditions, including substance use disorders; anxiety, stress-related, and somatoform disorders; mood disorders; schizophrenia, schizotypal, and delusional disorders; self-harm; and childhood-onset behavioral disorders.

Relatively few studies have examined the association between heat and ED visits for mental health. Regional studies conducted in many cities and countries, including in California,^9,31^ Southern California,^32^ and New York^10^ in the US; Adelaide, Australia^10^; Paris, France^33^; Tel Aviv, Israel^34^; the Baix Camp and Tarragona region of Spain^35^; and Canada,^36^ have found an increasing number of ED visits for a variety of mental health conditions associated with increasing temperatures. Another study based in Barcelona, Spain, found no association between heat and ED visits in the general population but did find elevated risk factors among patients with psychiatric histories, as well as more alcohol and drug misuse, during an extreme heat wave in 2003.^37^ However, these studies often rely on data from local hospitals or regional health care utilization data, potentially limiting the generalizability of results. By comparison, our findings extend the previous work by examining the implications of temperature for ED utilization for mental health conditions among adults with health insurance across the entire contiguous US.

In addition, we examined the potential for elevated rates of ED visits associated with any mental health diagnosis among different age groups as well as among men vs women and within different US regions. We found no evidence of differential associations between temperature and mental health stratified by age groups, which stands in contrast to previous findings.^10^ We also found that the rate of ED visits on days of extreme heat was higher among men vs women, a different result from past work.^31^ We also found higher rates of ED visits in the US Northwest, Northeast, and Midwest, a regional analysis that has not been previously conducted for mental health outcomes in the US. This finding may suggest that there is an increased risk of adverse mental health outcomes in regions of the US that are less well adapted to heat (ie, where adaptive measures such as air conditioning may be less prevalent compared with areas, such as the Southeastern and Southwestern US, that have historically experienced higher temperatures^38^).

There are several potential pathways by which heat may exacerbate mental health conditions. Exogenous stressors are well known to exacerbate existing mental health conditions. Our finding that heat was associated with a similar increase in the rate of ED visits for a variety of different mental health conditions is consistent with the hypothesis that heat is an external stressor that is not specific to any given mental health condition. One etiological mechanism may be disrupted sleep during periods of high ambient temperature, which may be associated with adverse mental health outcomes.^39^ Daytime discomfort or irritation owing to elevated temperature may be a stressor that exacerbates preexisting conditions. Another biological pathway may be the increase in hopelessness, maladaptive anxiety, and stress attributable to the anticipation of climate change and associated extreme events.^40,41,42,43^ In addition, on warmer days, patients may visit the ED to seek relief from high temperatures. Heat could also affect opening hours of other health care facilities, which could be associated with an increase in ED visits. These and other social and health care system factors might explain elevated ED visits on days of extreme temperature.

### Strengths and Limitations

This study has strengths. To our knowledge, it is the largest and most comprehensive analysis of daily ambient temperature associated with ED visits for mental health diagnoses among adults aged 18 years or older across the contiguous US. Because we focused on ED visits, which represent clinically meaningful exacerbations of mental health conditions, we were able to assess the costliest interactions between temperature and mental health both at the individual level and from the perspective of the health care system. With such a large data set, we were able to explore the consequences of temperature on a wide range of illnesses associated with adverse mental health outcomes, filling an important gap in the existing literature. The current analysis focused on the warm season; future work is needed to further characterize the implications of temperature for mental health outcomes during cold seasons. We were also able to identify some strata of the population that may have more risk factors for adverse mental health outcomes owing to extreme heat. Additional studies are needed to identify other populations that may be at greater risk for adverse outcomes and to gain insights into the pathophysiologic mechanisms underlying the observed associations in an effort to identify effective strategies to prevent adverse mental health outcomes.

The association between elevated ambient temperature and an increased rate of ED visits for specific mental health conditions, such as substance use disorders, may be of particular relevance to mental health practitioners and public health officials during periods of extreme heat. It is possible that the association between extreme heat and exacerbation of symptoms for many mental and behavioral disorders is not limited to ED visits but may also include a broader group of people with mental health conditions that may not require emergency care. During and following periods of high temperature, mental health and emergency care practitioners may consider increasing capacity to provide necessary mental health services. This consideration is particularly important given the potential for climate change to increase both the frequency and severity of extreme temperatures,^29^ which may further increase demand for clinical services related to mental health and may also lead to increased direct emotional responses such as anxiety.^40^

This study also has limitations. First, although our use of the case-crossover study presented some advantages, there are some limitations to causal interpretation of the effect size estimates. This study design is appropriate when exposure is intermittent, the implications for the risk of outcome are immediate, and the outcome itself is abrupt—a series of general criteria that suit our study.^23,24^ We estimate that potential causes of bias within our study design would bias the results toward the null. For example, we used the population-weighted mean daily maximum temperature as a proxy for personal heat exposure, potentially leading to some exposure misclassification. However, we expect that this exposure misclassification would be nondifferential and on average tend to bias our results toward the null. In addition, there may be unmeasured time-varying confounders, including time spent outdoors and activity levels, which we anticipate would be nondifferential and on average bias our results toward the null.

Second, we did not consider other meteorological characteristics, such as precipitation or cloud cover, either of which may alter mental health.^44,45^ However, given that warm-season days with precipitation or substantial cloud cover are generally cooler than what would be observed under equivalent clear-sky conditions, we expect that any confounding by these elements (if present) would have biased our estimates toward the null hypothesis of no association between extreme heat and an increase in ED visits for mental health conditions.

Third, our study is based on health care utilization data, and given that it specifically focused on ED visits, we anticipate that the mental health diagnoses included in this study likely represent the most severe presentations. The less severe outcomes associated with increasing temperature are an area for future research.

Fourth, use of deidentified medical claims data limits the information available on individual-level characteristics; data on race and ethnicity, individual markers of socioeconomic means, occupation, and time-activity patterns were not available. Although these factors cannot confound the results because of the use of the study’s design, we were not able to comprehensively assess individual-level risk factors.

Fifth, our data are limited to individuals with commercial health insurance or Medicare Advantage (ie, data do not include recipients of Medicaid health coverage for individuals with a low income or Medicare without supplemental plans, hence likely skewing of the sample toward wealthier socioeconomic status), potentially limiting the generalizability of our results.

## Conclusions

Results of this case-crossover study suggest that there was an association between elevated ambient temperature and ED visits for any mental health condition and for specific mental health diagnoses. This finding could aid clinicians who provide mental health services in preparing for increases in health service needs when high ambient temperature is anticipated. Further research could investigate the implications of sustained periods of extreme heat (heat waves) for health outcomes and continue to investigate the association among different populations. In addition, future work could characterize the implications of elevated temperatures during cold periods for mental health outcomes and the consequences of additional meteorological characteristics and multiple extreme weather events that may occur with elevated ambient temperature or may be triggered by periods of extreme heat.

## Notes

Supplement. **eTable.** CCS Codes and Corresponding *ICD-9/ICD-10* Codes. **eFigure 1.** Time Course for Extreme Heat Exposure Response Curve. **eFigure 2.** Sensitivity Analysis Results and Time Course for Composite Mental Health End Point. **eFigure 3.** Time Course for Cause-Specific Mental Health Emergency Department Visits. **eFigure 4.** Incidence Rate Ratio of Emergency Department Visits for 95th Percentile of Temperature vs Optimal Temperature Among Subgroups, and Heterogeneity Tests. **eAppendix.** Sample R Code for Analysis.
