# Supplementary material for: Association Between Ambient Heat and Risk of Emergency Department Visits for Mental Health Among US Adults, 2010 to 2019
Source: JAMA Psychiatry. 2022 Feb 23;79(4):341–9. doi: 10.1001/jamapsychiatry.2021.4369 (PMC8867392; doi:10.1001/jamapsychiatry.2021.4369)
Supplement: Supplement. — eTable. CCS Codes and Corresponding ICD-9/ICD-10 Codes. eFigure 1. Time Course for Extreme Heat Exposure Response Curve. eFigure 2. Sensitivity Analysis Results and Time Course for Composite Mental Health End Point. eFigure 3. Time Course for Cause-Specific Mental Health Emergency Department Visits. eFigure 4. Incidence Rate Ratio of Emergency Department Visits for 95th Percentile of Temperature vs Optimal Temperature Among Subgroups, and Heterogeneity Tests. eAppendix. Sample R Code for Analysis. [file jamapsychiatry-e214369-s001.pdf]

Supplementary Online Content

Nori-Sarma A, Sun S, Sun Y, et al. Association between ambient heat and risk of emergency department visits for mental health among US adults, 2010 to 2019. JAMA Psychiatry. Published online February 23, 2022. doi:10.1001/jamapsychiatry.2021.4369

eTable. CCS Codes and Corresponding ICD-9/ICD-10 Codes
eFigure 1. Time Course for Extreme Heat Exposure Response Curve
eFigure 2. Sensitivity Analysis Results and Time Course for Composite Mental Health End Point
eFigure 3. Time Course for Cause-Specific Mental Health Emergency Department Visits
eFigure 4. Incidence Rate Ratio of Emergency Department Visits for 95th Percentile of Temperature vs Optimal Temperature Among Subgroups, and Heterogeneity Tests
eAppendix. Sample R Code for Analysis

This supplementary material has been provided by the authors to give readers additional information about their work.

© 2022 Nori-Sarma et al. *JAMA Psychiatry.*

eTable: CCS Codes and Corresponding *ICD 9 / ICD 10* codes

CCS Code	ICD9 Code	ICD10 Code
650	3090, 3091, 30922, 30923, 30924, 30928, 30929, 3093, 3094, 30982, 30983, 30989, 3099	F4320, F4321, F4322, F4323,<br>F4324, F4325, F4329, F438, F439
651	29384, 30000, 30001, 30002, 30009, 30010, 30020, 30021, 30022, 30023, 30029, 3003, 3005, 30089, 3009, 3080, 3081, 3082, 3083, 3084, 3089, 30981, 3130, 3131, 31321, 31322, 3133, 31382, 31383	F064, F4000, F4001, F4002, F4010, F4011, F40210, F40218, F40220, F40228, F40230, F40231, F40232, F40233, F40240, F40241, F40242, F40243, F40248, F40290, F40291, F40298, F408, F409, F410, F411, F413, F418, F419, F42, F422, F423, F424, F428, F429, F430, F4310, F4311, F4312, F488, F489, R452, R453, R454, R455, R456, R457, R4581, R4582, R4583, R4584
652	31200, 31201, 31202, 31203, 31210, 31211, 31212, 31213, 31220, 31221, 31222, 31223, 3124, 3128, 31281, 31282, 31289, 3129, 31381, 31400, 31401, 3141, 3142, 3148, 3149	F900, F901, F902, F908, F909, F910, F911, F912, F913, F918, F919, R460, R461, R462, R463, R464, R465, R466, R467, R4681, R4689
654	3070, 3079, 31500, 31501, 31502, 31509, 3151, 3152, 31531, 31532, 31534, 31535, 31539, 3154, 3155, 3158, 3159, 317, 3180, 3181, 3182, 319, V400, V401	F70, F71, F72, F73, F78, F79, F800, F801, F804, F8081, F8082, F8089, F809, F810, F812, F8181, F8189, F819, F82, F88, F89, F985, R4183, R480
655	29900, 29901, 29910, 29911, 29980, 29981, 29990, 29991, 30720, 30721, 30722, 30723, 3073, 3076, 3077, 30921, 31323, 31389, 3139	F642, F840, F842, F843, F845, F848, F849, F930, F938, F939, F940, F941, F942, F948, F949, F950, F951, F952, F958, F959, F980, F981, F9821, F9829, F983, F984, F988, F989
657	29383, 29600, 29601, 29602, 29603, 29604, 29605, 29606, 29610, 29611, 29612, 29613, 29614, 29615, 29616, 29620, 29621, 29622, 29623, 29624, 29625, 29626, 29630, 29631, 29632, 29633, 29634, 29635, 29636, 29640, 29641, 29642, 29643, 29644 29645, 29646, 29650, 29651, 29652, 29653, 29654, 29655, 29656, 29660, 29661, 29662, 29663, 29664, 29665,	F0630-F0634, F3011-F3013, F302-F304, F308-F310, F3110-F3113, F312, F3130-F3132, F314, F315, F3160-F3164, F3170-F3178, F3181, F3189, F319-F325, F328, F3281, F3289, F329-F333, F3340-F3342, F338-F341, F348, F3481, F3489, F349, F39, R4586

© 2022 Nori-Sarma et al. *JAMA Psychiatry.*

	29666, 2967, 29680, 29681, 29682, 29689, 29690, 29699, 3004, 311	
658	3010 30110 30111 30112 30113 30120 30121 30122 3013 3014 30150 30151 30159 3016 3017 30181 30182 30183 30184 30189 3019	F600-F607, F6081, F6089, F609, F69
659	29381 29382 29500 29501 29502 29503 29504 29505 29510 29511 29512 29513 29514 29515 29520 29521 29522 29523 29524 29525 29530 29531 29532 29533 29534 29535 29540 29541 29542 29543 29544 29545 29550 29551 29552 29553 29554 29555 29560 29561 29562 29563 29564 29565 29570 29571 29572 29573 29574 29575 29580 29581 29582 29583 29584 29585 29590 29591 29592 29593 29594 29595 2970 2971 2972 2973 2978 2979 2980 2981 2982 2983 2984 2988 2989	F060, F062, F200-F203, F205, F2081, F2089, F209, F21-F24, F250, F251, F258, F259, F28, F29
660	2910 2911 2912 2913 2914 2915 2918 29181 29182 29189 2919 30300 30301 30302 30303 30390 30391 30392 30393 30500 30501 30502 30503 3575 4255 5353 53530 53531 5710 5711 5712 5713 76071 9800	F1010, F1011, F10120, F10121, F10129, F1014, F10150, F10151, F10159, F10180-F10182, F10188, F1019-F1021, F10220, F10221, F10229-F10232, F10239, F1024, F10250, F10251, F10259, F1026, F1027, F10280-F10282, F10288, F1029, F10920, F10921, F10929, F1094, F10950, F10951, F10959, F1096, F1097, F10980-F10982, F10988, F1099, G621, I426, K2920, K2921, K700, K7010, K7011, K702, K7030, K7031, K7040, K709, O99310-O99315, P043, Q860
661	2920 29211 29212 2922 29281 29282 29283 29284 29285 29289 2929 30400 30401 30402 30403 30410 30411 30412 30413 30420 30421 30422 30423 30430 30431 30432 30433 30440 30441 30442 30443 30450 30451 30452 30453 30460 30461 30462 30463 30470 30471 30472 30473 30480 30481 30482 30483 30490 30491 30492 30493 30520 30521 30522 30523 30530 30531 30532 30533 30540 30541 30542 30543 30550	F1110, F1111, F11120-F11122, F11129, F1114, F11150, F11151, F11159, F11181, F11182, F11188, F1119-F1121, F11220-F11222, F11229, F1123, F1124, F11250-F11251, F11259, F11281, F11282, F11288, F1129, F1190, F11920-F11922, F11929, F1193, F1194, F11950, F11951, F11959, F11981, F11982, F11988, F1199, F1210, F1211, F12120-F12122, F12129, F12150-F12151, F12159, F12180, F12188, F1219-F1221, F12220-F12222, F12229, F1223, F12250, F12251, F12259, F12280, F12288, F1229, F1290, F12920-F12922, F12929, F1293, F12950-F12951, F12959, F12980, F12988, F1299, F1310, F1311, F13120-F13121, F13129, F1314, F13150, F13151, F13159, F13180,

© 2022 Nori-Sarma et al. *JAMA Psychiatry.*

	30551 30552 30553 30560 30561 30562 30563 30570 30571 30572 30573 30580 30581 30582 30583 30590 30591 30592 30593 64830 64831 64832 64833 64834 65550 65551 65553 76072 76073 76075 7795 96500 96501 96502 96509 V6542	F13181, F13182, F13188, F1319-F1321, F13220, F13221, F13229-F13232, F13239, F1324, F13250, F13251, F13259, F1326, F1327, F13280-F13282, F13288, F1329, F1390, F13920, F13921, F13929-F13932, F13939, F1394, F13950, F13951, F13959, F1396, F1397, F13980-F13982, F13988, F1399, F1410, F1411, F14120-F14122, F14129, F1414, F14150, F14151, F14159, F14180-F14182, F14188, F1419-F1421, F14220-F14222, F14229, F1423, F1424, F14250, F14251, F14259, F14280-F14282, F14288, F1429, F1490, F14920-F14922, F14929, F1494, F14950-, F14951, F14959, F14980-F14982, F14988, F1499, F1510, F1511, F15120-F15122, F15129, F1514, F15150, F15151, F15159, F15180-F15182, F15188, F1519-F1521, F15220-F15222, F15229, F1523, F1524, F15250, F15251, F15259, F15280-F15282, F15288, F1529, F1590, F15920-F15922, F15929, F1593, F1594, F15950, F15951, F15959, F15980-F15982, F15988, F1599, F1610, F1611, F16120-F16122, F16129, F1614, F16150, F16151, F16159, F16180, F16183, F16188, F1619-F1621, F16220, F16221, F16229, F1624, F16250, F16251, F16259, F16280, F16283, F16288, F1629, F1690, F16920, F16921, F16929, F1694, F16950-F16951, F16959, F16980, F16983, F16988, F1699, F17200, F17201, F17203, F17208-F17211, F17213, F17218-F17221, F17223, F17228, F17229, F17290, F17291, F17293, F17298, F17299, F1810, F1811, F18120, F18121, F18129, F1814, F18150, F18151, F18159, F1817, F18180, F18188, F1819-F1821, F18220, F18221, F18229, F1824, F18250, F18251, F18259, F1827, F18280, F18288, F1829, F1890, F18920, F18921, F18929, F1894, F18950, F18951, F18959, F1897, F18980, F18988, F1899, F1910, F1911, F19120-F19122, F19129, F1914, F19150, F19151, F19159, F1916, F1917, F19180-F19182, F19188, F1919-F1921, F19220-F19222, F19229-F19232, F19239, F1924, F19250, F19251, F19259, F1926, F1927, F19280-F19282, F19288, F1929, F1990, F19920-F19922, F19929-F19932, F19939, F1994, F19950, F19951, F19959, F1996, F1997, F19980-F19982, F19988, F1999, F550-F554, F558, O355XX0-O355XX5, O355XX9, O99320-O99325, P0441, P0449, P961, P962, T400X1A, T400X1D, T400X1S, T400X3A, T400X3D, T400X3S, T400X4A, T400X4D, T400X4S, T400X5A, T400X5D, T400X5S, T400X6A, T400X6D, T400X6S, T401X1A, T401X1D, T401X1S, T401X3A, T401X3D, T401X3S, T401X4A, T401X4D, T401X4S, T401X5A, T401X5D, T401X5S, T405X1A, T405X1D, T405X1S, T405X3A, T405X3D, T405X3S, T405X4A, T405X4D, T405X4S, T405X5A, T405X5D, T405X5S, T405X6A, T405X6D, T405X6S, T407X1A, T407X1D, T407X1S, T407X3A, T407X3D, T407X3S, T407X4A, T407X4D, T407X4S, T407X5A, T407X5D, T407X5S, T408X1A, T408X1D,

© 2022 Nori-Sarma et al. *JAMA Psychiatry.*

		T408X1S, T408X3A, T408X3D, T408X3S, T408X4A, T408X4D, T408X4S, T408X5A, T408X5D, T408X5S, T40901A, T40901D, T40901S, T40903A, T40903D, T40903S, T40904A, T40904D, T40904S, T40905A, T40905D, T40905S, T40906A, T40906D, T40906S, T40991A, T40991D, T40991S, T40994A, T40994D, T40994S, T40995A, T40995D, T40995S, T40996A, T40996D, T40996S
662	E9500 E9501 E9502 E9503 E9504 E9505 E9506 E9507 E9508 E9509 E9510 E9511 E9518 E9520 E9521 E9528 E9529 E9530 E9531 E9538 E9539 E954 E9550 E9551 E9552 E9553 E9554 E9555 E9556 E9557 E9559 E956 E9570 E9571 E9572 E9579 E9580 E9581 E9582 E9583 E9584 E9585 E9586 E9587 E9588 E9589 E959 V6284	R45851, T1491, T1491XA, T1491XD, T1491XS, (T360X2A, T360X2D, T360X2S)- (T375X2A, T375X2D, T375X2S), (T378X2A, T378X2D, T378X2S)- (T387X2A, T387X2D, T387X2S), T38802A, T38802D, T38802S, T38812A, T38812D, T38812S, T38892A, T38892D, T38892S, T38902A, T38902D, T38902S, T38992A, T38992D, T38992S, T39012A, T39012D, T39012S, T39092A, T39092D, T39092S, T391X2A, T391X2D, T391X2S, T392X2A, T392X2D, T392X2S, T39312A, T39312D, T39312S, T39392A, T39392D, T39392S, T394X2A, T394X2D, T394X2S, T398X2A, T398X2D, T398X2S, T3992XA, T3992XD, T3992XS, (T400X2A, T400X2D, T400X2S) - (T405X2A, T405X2D, T405X2S), T40602A, T40602D, T40602S, T40692A, T40692D, T40692S, (T407X2A, T407X2D, T407X2S) - (T411X2A, T411X2D, T411X2S), T41202A, T41202D, T41202S, T41292A, T41292D, T41292S, T413X2A, T413X2D, T413X2S, T4142XA, T4142XD, T4142XS, T415X2A, T415X2D, T415X2S, (T420X2A, T420X2D, T420X2S) - (T426X2A, T426X2D, T426X2S), T4272XA, T4272XD, T4272XS, T428X2A, T428X2D, T428X2S, T43012A, T43012D, T43012S, T43022A, T43022D, T43022S, T431X2A, T431X2D, T431X2S, T43202A, T43202D, T43202S, T43212A, T43212D, T43212S, T43222A, T43222D, T43222S, T43292A, T43292D, T43292S, T433X2A, T433X2D, T433X2S, T434X2A, T434X2D, T434X2S, T43502A, T43502D, T43502S , T43592A, T43592D, T43592S, T43602A, T43602D, T43602S, T43612A, T43612D, T43612S, T43622A, T43622D, T43622S, T43632A, T43632D, T43632S, T43692A, T43692D, T43692S, T438X2A, T438X2D, T438X2S, T4392XA, T4392XD, T4392XS, (T440X2A, T440X2D, T440X2S) - (T448X2A, T448X2D, T448X2S), T44902A, T44902D, T44902S, T44992A, T44992D, T44992S, (T450X2A, T450X2D, T450X2S) - (T454X2A, T454X2D, T454X2S), T45512A, T45512D, T45512S, T45522A, T45522D, T45522S, T45602A, T45602D, T45602S, T45612A, T45612D, T45612S, T45622A, T45622D, T45622S, T45692A, T45692D, T45692S, T457X2A, T457X2D, T457X2S, T458X2A, T458X2D, T458X2S, T4592XA, T4592XD, T4592XS, (T460X2A, T460X2D, T460X2S) - (T468X2A, T468X2D, T468X2S),

© 2022 Nori-Sarma et al. *JAMA Psychiatry.*

		T46902A, T46902D, T46902S, T46992A, T46992D, T46992S, (T470X2A, T470X2D, T470X2S) - (T481X2A, T481X2D, T481X2S), T48202A, T48202D, T48202S, T48292A, T48292D, T48292S, (T483X2A, T483X2D, T483X2S) - (T486X2A, T486X2D, T486X2S), T48902A, T48902D, T48902S, T48992A, T48992D, T48992S, (T490X2A, T490X2D, T490X2S) - (T498X2A, T498X2D, T498X2S), T4992XA, T4992XD, T4992XS, (T500X2A, T500X2D, T500X2S) - (T508X2A, T508X2D, T508X2S), T50902A, T50902D, T50902S, T50992A, T50992D, T50992S, T50A12A, T05A12D, T50A12S, T50A22A, T50A22D, T50A22S, T50A92A, T50A92D, T50A92S, T50B12A, T50B12D, T50B12S, T50B92A, T50B92D, T50B92S, T50Z12A, T50Z12D, T50Z12S, T50Z92A, T50Z92D, T50Z92S, (T510X2A, T510X2D, T510X2S) - (T513X2A, T513X2D, T513X2S), T518X2A, T518X2D, T518X2S, T5192XA, T5192XD, T5192XS, (T520X2A, T520X2D, T520X2S) - (T524X2A, T524X2D, T524X2S), T528X2A, T528X2D, T528X2S, T5292XA, T5292XD, T5292XS, (T530X2A, T530X2D, T530X2S) - (T537X2A, T537X2D, T537X2S), T5392XA, T5392XD, T5392XS, (T540X2A, T540X2D, T540X2S) - (T543X2A, T543X2D, T543X2S), T5492XA, T5492XD, T5492XS, T550X2A, T550X2D, T550X2S, T551X2A, T551X2D, T551X2S, (T560X2A, T560X2D, T560X2S) - (T567X2A, T567X2D, T567X2S), T56812A, T56812D, T56812S, T56892A, T56892D, T56892S, T5692XA, T5692XD, T5692XS, (T570X2A, T570X2D, T570X2S) - (T573X2A, T573X2D, T573X2S), T578X2A, T578X2D, T578X2S, (T5792XA, T5792XD, T5792XS) - (T5812XA, T5812XD, T5812XS), T582X2A, T582X2D, T582X2S, T588X2A, T588X2D, T588X2S, T5892XA, T5892XD, T5892XS, (T590X2A, T590X2D, T590X2S) - (T597X2A, T597X2D, T597X2S), T59812A, T59812D, T59812S, T59892A, T59892D, T59892S, T5992XA, T5992XD, T5992XS, (T600X2A, T600X2D, T600X2S) - (T604X2A, T604X2D, T604X2S), T608X2A, T608X2D, T608X2S, T6092XA, T6092XD, T6092XS, T6102XA, T6102XD, T6102XS, T6112XA, T6112XD, T6112XS, T61772A, T61772D, T61772S, T61782A, T61782D, T61782S, T618X2A, T618X2D, T618X2S, T6192XA, T6192XD, T6192XS, (T620X2A, T620X2D, T620X2S) - (T622X2A, T622X2D, T622X2S), T628X2A, T628X2D, T628X2S, T6292XA, T6292XD, T6292XS, T63002A, T63002D, T63002S, T63012A, T63012D, T63012S T63022A, T63022D, T63022S, T63032A, T63032D, T63032S, T63042A, T63042D, T63042S, T63062A, T63062D, T63062S, T63072A, T63072D, T63072S, T63082A, T63082D, T63082S, T63092A, T63092D, T63092S, T63112A, T63112D, T63112S, T63122A, T63122D, T63122S, T63192A, T63192D, T63192S, T632X2A, T632X2D, T632X2S, T63302A,

© 2022 Nori-Sarma et al. *JAMA Psychiatry.*

		T63302D, T63302S, T63312A, T63312D, T63312S, T63322A, T63322D, T63322S, T63332A, T63332D, T63332S, T63392A, T63392D, T63392S, T63412A, T63412D, T63412S, T63422A, T63422D, T63422S, T63432A, T63432D, T63432S, T63442A, T63442D, T63442S, T63452A, T63452D, T63452S, T63462A, T63462D, T63462S, T63482A, T63482D, T63482S, T63512A, T63512D, T63512S, T63592A, T63592D, T63592S, T63612A, T63612D, T63612S, T63622A, T63622D, T63622S, T63632A, T63632D, T63632S, T63692A, T63692D, T63692S, T63712A, T63712D, T63712S, T63792A, T63792D, T63792S, T63812A, T63812D, T63812S, T63822A, T63822D, T63822S, T63832A, T63832D, T63832S, T63892A, T63892D, T63892S, T6392XA, T6392XD, T6392XS, T6402XA, T6402XD, T6402XS, T6482XA, T6482XD, T6482XS, T650X2A, T650X2D, T650X2S, T651X2A, T651X2D, T651X2S, T65212A, T65212D, T65212S, T65222A, T65222D, T65222S, T65292A, T65292D, T65292S, (T653X2A, T653X2D, T653X2S) - (T656X2A, T656X2D, T656X2S), T65812A, T65812D, T65812S, T65822A, T65822D, T65822S, T65832A, T65832D, T65832S, T65892A, T65892D, T65892S, T6592XA, T6592XD, T6592XS, T71112A, T71112D, T71112S, T71122A, T71122D, T71122S, T71132A, T71132D, T71132S, T71152A, T71152D, T71152S, T71162A, T71162D, T71162S, T71192A, T71192D, T71192S, T71222A, T71222D, T71222S, T71232A, T71232D, T71232S, (X710XXA, X710XXD, X710XXS) - (X713XXA, X713XXD, X713XXS), X718XXA, X718XXD, X718XXS, X719XXA, X719XXD, X719XXS, X72XXXA, X72XXXD, X72XXXS, (X730XXA, X730XXD, X730XXS) - (X732XXA, X732XXD, X732XXS), X738XXA, X738XXD, X738XXS, X739XXA, X739XXD, X739XXS, X7401XA, X7401XD, X7401XS, X7402XA, X7402XD, X7402XS, X7409XA, X7409XD, X7409XS, X748XXA, X748XXD, X748XXS, X749XXA, X749XXD, X749XXS, X75XXXA, X75XXXD, X75XXXS, X76XXXA, X76XXXD, X76XXXS, (X770XXA, X770XXD, X770XXS) - (X773XXA, X773XXD, X773XXS), (X778XXA, X778XXD, X778XXS) - (X782XXA, X782XXD, X782XXS), X788XXA, X788XXD, X788XXS, X789XXA, X789XXD, X789XXS, X79XXXA, X79XXXD, X79XXXS, X80XXXA, X80XXXD, X80XXXS, X810XXA, X810XXD, X810XXS, X811XXA, X811XXD, X811XXS, X818XXA, X818XXD, X818XXS, (X820XXA, X820XXD, X820XXS) - (X822XXA, X822XXD, X822XXS), X828XXA, X828XXD, X828XXS, (X830XXA, X830XXD, X830XXS) - (X832XXA, X832XXD, X832XXS), X838XXA, X838XXD, X838XXS, Z915

© 2022 Nori-Sarma et al. *JAMA Psychiatry.*

670	29389 2939 30011 30012 30013 30014 30015 30016 30019 3006 3007 30081 30082 3021 3022 3023 3024 30250 30251 30252 30253 3026 30270 30271 30272 30273 30274 30275 30276 30279 30281 30282 30283 30284 30285 30289 3029 3060 3061 3062 3063 3064 30650 30651 30652 30653 30659 3066 3067 3068 3069 3071 30740 30741 30742 30743 30744 30745 30746 30747 30748 30749 30750 30751 30752 30753 30754 30759 30780 30781 30789 3101 316 64840 64841 64842 64843 64844 V402 V403 V4031 V4039 V409 V673	F061, F068, F440-F442, F444-F447, F4481, F4489, F449-F451, F4520-F4522, F4529, F4541, F4542, F458, F459, F481, F5000-F5002, F502, F508, F5081, F5082, F5089, F509, F5101-F5105, F5109, F5111-F5113, F5119, F513-F515, F518-F521, F5221, F5222, F5231, F5232, F524-F526, F528, F529, F53, F530, F531, F54, F59, F640, F641, F648-F654, F6550-F6552, F6581, F6589, F659, F66, F6810-F6813, F688, F68A, F99, O906, R37, R4589, Z87890, Z9183

© 2022 Nori-Sarma et al. *JAMA Psychiatry.*

eFigure 1. Time Course for Extreme Heat Exposure Response Curve

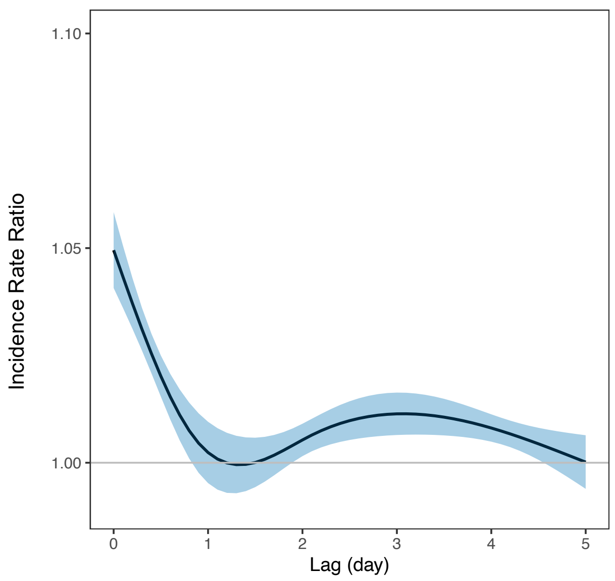

© 2022 Nori-Sarma et al. *JAMA Psychiatry.*

eFigure 2. Sensitivity Analysis Results and Time Course for Composite Mental Health End Point

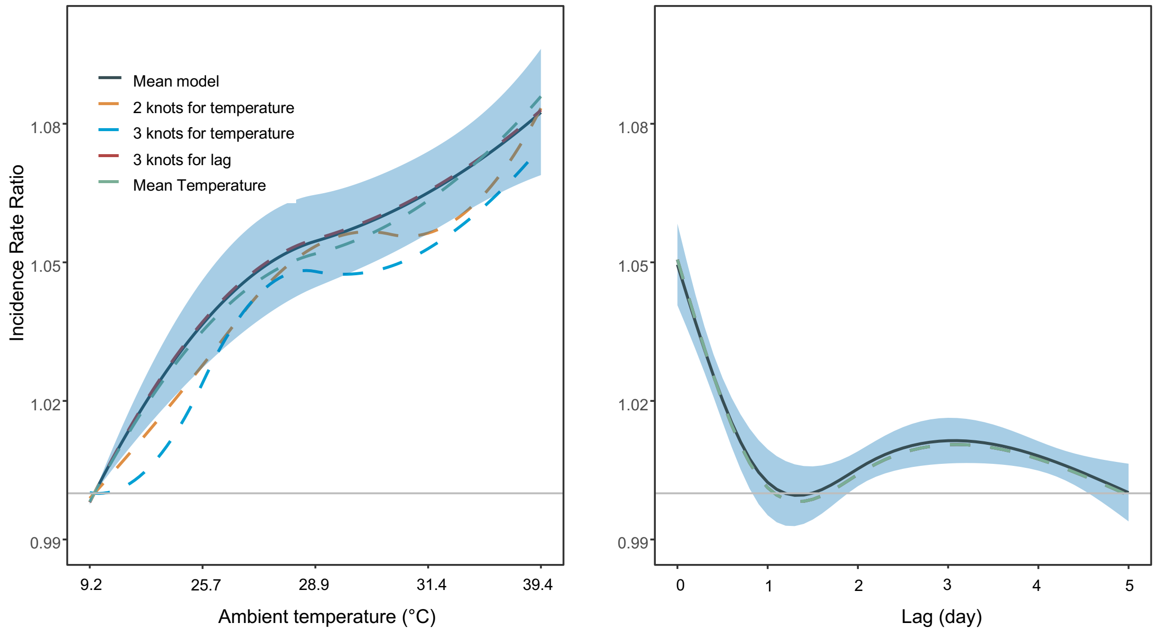

© 2022 Nori-Sarma et al. *JAMA Psychiatry.*

eFigure 3. Time Course for Cause-Specific Mental Health Emergency Department Visits

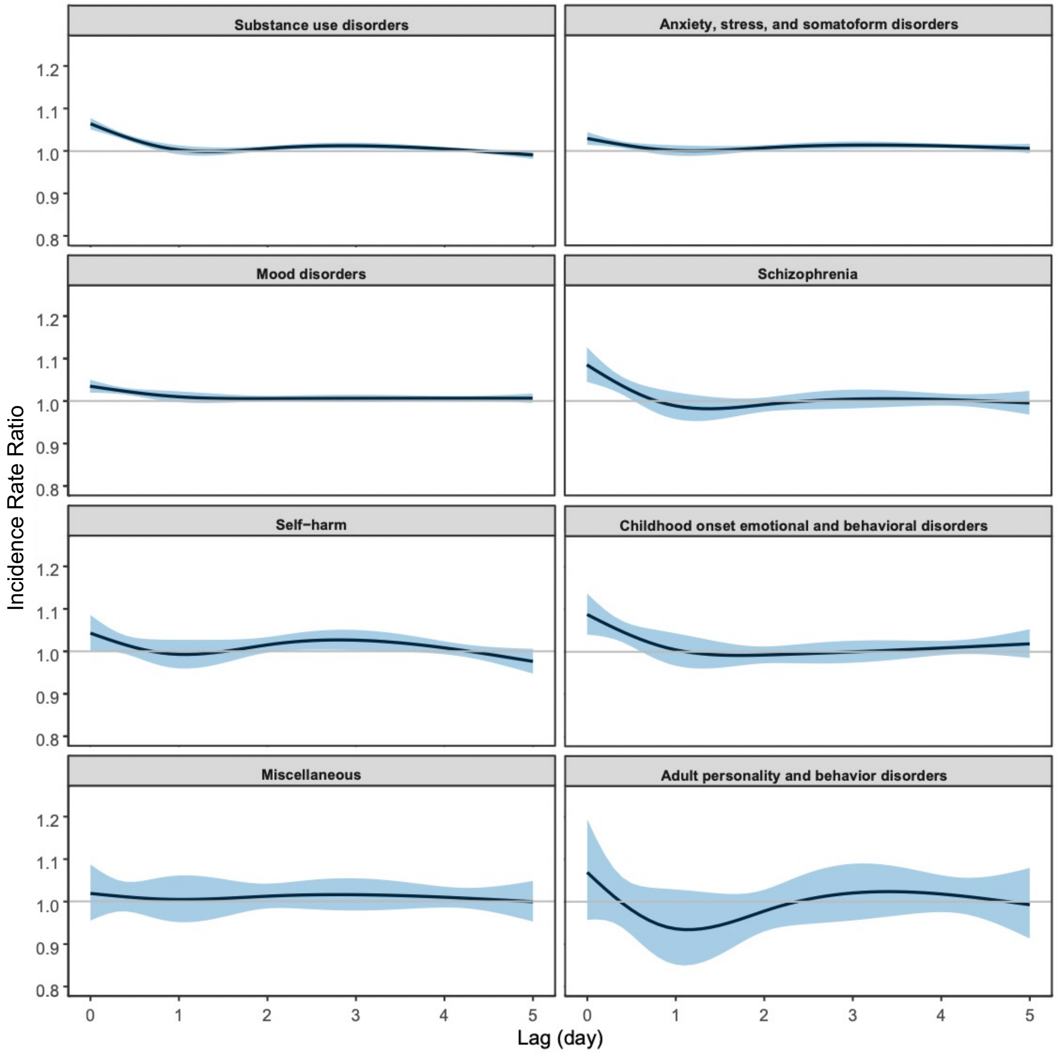

© 2022 Nori-Sarma et al. *JAMA Psychiatry.*

eFigure 4. Incidence Rate Ratio of Emergency Department Visits for 95th Percentile of Temperature vs Optimal Temperature Among Subgroups, and Heterogeneity Tests

Characteristics	Subgroup	Incidence Rate Ratio (95% CI) 95th vs. Optimal Temperature	p-value for Heterogeneity
	Overall	1.08 (1.07, 1.09)	
Age	18-26 y	1.07 (1.04, 1.10)	0.273
	27-44 y	1.08 (1.06, 1.10)	
	45-64 y	1.09 (1.07, 1.11)	
	65+ y	1.06 (1.04, 1.09)	
Sex	Male	1.10 (1.08, 1.12)	0.006
	Female	1.06 (1.05, 1.08)	
NCA4 Region	Northeast	1.10 (1.07, 1.13)	0.003
	Southeast	1.05 (1.03, 1.07)	
	Midwest	1.11 (1.09, 1.13)	
	Northern Great Plains	1.10 (1.00, 1.21)	
	Northwest	1.12 (1.03, 1.21)	
	Southern Great Plains	1.06 (1.03, 1.10)	
	Southwest	1.08 (1.04, 1.12)	

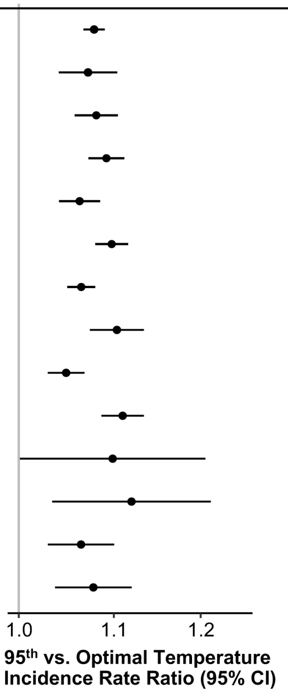

eAppendix. Sample R Code for Analysis

```
####################################################################################
# Sample R code for the analysis in:
# "Ambient heat and risk of emergency department visits for
# mental health among adults in the United States, 2010-2019"
# JAMA Psychiatry 2021
####################################################################################
library(tidyverse);library(readr);library(lubridate)
library(splines);library(dlnm);library(survival)

# function for dose-response curve
dset <- function(var, dis){
  dat <- claim2010_2019 %>% select(optum_lab_id, stay_start, county_fips,{{var}}) %>%
```

© 2022 Nori-Sarma et al. *JAMA Psychiatry.*

```
  filter({{var}}==1) %>%
  distinct(optum_lab_id, stay_start, .keep_all=TRUE) %>% ungroup() %>%
  select(-optum_lab_id) %>%
  group_by(stay_start, county_fips) %>% add_tally() %>%
  distinct(stay_start, county_fips, n) %>% ungroup() %>%
  mutate(strata=1:nrow(.))
# load('Z:\\darren\\heat_adults\\tmean.rda')

 dat <- left_join(dat, data, by='stay_start')
 dat <- left_join(dat, prism2010_2019, by=c('county_fips', 'control_date'))

 dat <- dat %>% ungroup() %>% select(-c(county_fips, stay_start, Tmax_F,lag6,lag7))

 basis <- crossbasis(dat[,c(6:11)],lag = 5, argvar = list(fun='bs',degree=2, knots=0.5),
                   arglag = list(knots=logknots(5, 2)))

 model <- clogit(case ~ basis + strata(strata)+ns(RHmean, 3)+holid, data=dat, weights = n,
               method = 'breslow')

 perct <- seq(0.01, 0.99, by = 0.01)
 pred <- crosspred(basis, model, cen=0.5, at= perct)
 pmin <- perct[which.min(pred$allfit)]

 pred <- crosspred(basis, model, cen=pmin, at=perct)

 conc_dis <- rownames_to_column(data.frame(allRRfit=pred$allRRfit, allRRlow=pred$allRRlow,
                                   allRRhigh=pred$allRRhigh),var = 'conc') %>%
  mutate(conc=as.numeric(conc))
```

© 2022 Nori-Sarma et al. *JAMA Psychiatry.*

```
  pred <- crosspred(basis, model, cen=pmin, bylag=0.1,
                   at=0.95)

  lag_dis=as_tibble_col(pred$matRRfit[1,], column_name = 'matRRfit') %>%
    mutate(lagday=names(pred$matRRfit[1,]),lag=parse_number(lagday),
           matRRlow=as_tibble_col(pred$matRRlow[1,]) %>% pull(value),
           matRRhigh=as_tibble_col(pred$matRRhigh[1,]) %>% pull(value))
  save(lag_dis, file = paste0(dis,'.rda'))
  save(conc_dis, file = paste0(dis,'.rda'))
}

dset(mental,'mental')



#-------------------------------------------------------------
# Function for relative risk
relatrisk <- function(var, num){
  dat <- claim2010_2019 %>% select(optum_lab_id, stay_start, county_fips,{{var}}) %>%
    filter({{var}}==num) %>%
    distinct(optum_lab_id, stay_start, .keep_all=TRUE) %>% ungroup() %>%
    select(-optum_lab_id) %>%
    group_by(stay_start, county_fips) %>% add_tally() %>%
    distinct(stay_start, county_fips, n) %>% ungroup() %>%
    mutate(strata=1:nrow(.))

  fips <- dat %>% distinct(county_fips) %>% pull(county_fips)
  prism <- prism2010_2019 %>% filter(county_fips %in% fips) %>%
    select(county_fips, Tmax_F)
  dlist <- lapply(fips, function(x) prism[prism$county_fips==x,])
```

© 2022 Nori-Sarma et al. *JAMA Psychiatry.*

```
names(dlist) <- fips

predper <- c(seq(1,99, by=0.1))

tmean <- rowMeans(sapply(dlist, function(x) quantile(x$Tmax_F, predper/100,na.rm = T)),
        na.rm = T)


dat <- left_join(dat, data, by='stay_start')

dat <- left_join(dat, prism2010_2019, by=c('county_fips', 'control_date'))


dat <- dat %>% ungroup() %>% select(-c(county_fips, stay_start, Tmax_F,lag6,lag7))




basis <- crossbasis(dat[,c(6:11)],lag = 5, argvar = list(fun='bs',degree=2, knots=0.5),
                    arglag = list(knots=logknots(5, 2)))


model <- clogit(case ~ basis + strata(strata)+ns(RHmean, 3)+holid, data=dat, weights = n,
       method = 'breslow')


perct <- seq(0.01, 0.99, by = 0.01)

pred <- crosspred(basis, model, cen=0.5, at= perct)

pmin <- perct[which.min(pred$allfit)]


pmin_value <- round((tmean[paste0(pmin*100,'.0%')]-32)*5/9,1)


extheat <- round((tmean['95.0%']-32)*5/9,1)

pred <- crosspred(basis, model, cen=pmin, at=c(0.85,0.95))


  c(length(fips),pmin, pmin_value,
extheat,pred$allfit[2],pred$allse[2],pred$allRRfit[2],pred$allRRlow[2],

   pred$allRRhigh[2])
```

© 2022 Nori-Sarma et al. *JAMA Psychiatry.*

```
}

Relatrisk(mental,1)
```

© 2022 Nori-Sarma et al. *JAMA Psychiatry.*
